# An Intelligent Automated Door Control System Based on a Smart Camera

**DOI:** 10.3390/s130505923

**Published:** 2013-05-10

**Authors:** Jie-Ci Yang, Chin-Lun Lai, Hsin-Teng Sheu, Jiann-Jone Chen

**Affiliations:** 1 Department of Electrical Engineering, National Taiwan University of Science and Technology, Taipei City 106, Taiwan; E-Mail: jjchen@ee.ntust.edu.tw; 2 Department of Communication Engineering, Oriental Institute of Technology, New Taipei City 220, Taiwan; E-Mail: fo001@mail.oit.edu.tw; 3 Department of Electrical Engineering, Tung-Nan University, New Taipei City 222, Taiwan; E-Mail: sheu@ee.ntust.edu.tw

**Keywords:** computer vision, curve fitting, Gaussian distribution, human detection, path analysis, power saving

## Abstract

This paper presents an innovative access control system, based on human detection and path analysis, to reduce false automatic door system actions while increasing the added values for security applications. The proposed system can first identify a person from the scene, and track his trajectory to predict his intention for accessing the entrance, and finally activate the door accordingly. The experimental results show that the proposed system has the advantages of high precision, safety, reliability, and can be responsive to demands, while preserving the benefits of being low cost and high added value.

## 1. Introduction

Automatic entrance/exit door control is widely used in public places such as grocery stores, businesses, transportation stations, airports, and wholesale department stores to eliminate the need of manually opening and closing actions. Contemporary sensor-based automatic door control technologies include infrared, ultrasonic/radio, or other wireless sensing methods. The first can be further divided into active and passive approaches. The active process emits infrared signals from the controller and captures the reflected signals to determine if there is any object close to the door. This approach is accurate and capable of identifying the position and the speed of the object but its high cost has made it less popular. The passive approach detects the infrared signals radiated by people and is the most widely used for being simple, effective, and low cost. The ultrasonic/radio approach, on the other hand, emits ultrasonic or radio waves to scan the environment and analyzes the returned signals for door access control.

Although these techniques are all successful in detecting objects, they are not capable of understanding the type and the intention of the objects. For instance, a puppy or a passing pedestrian may accidentally trigger the door and cause a false opening action. Frequent false action is not only annoying, and results in air conditioning energy waste, but also reduces equipment lifetime. This calls for the need of an automatic door control system based on the detection and intention analysis of people.

In this paper, door control is based on the confirmation that the detected object is indeed a human and the corresponding movement trajectory also indicates that he/she has the intention to go through the entrance. Furthermore, an infrared function has been added to prevent people from being trapped by the door before they leave the passage. In addition, the captured images can also be saved for other applications such as customer analysis and crime investigation.

This paper is organized as follows: Section 2 describes the system design concept and related theoretical basis. The corresponding hardware implementation architecture and the experimental results, as well as the performance analysis and discussions, on various field tests are described in Section 3. Finally, the conclusion and future work are made in the last section.

## 2. Design Concept and Principal Theory

**Figure 1.**
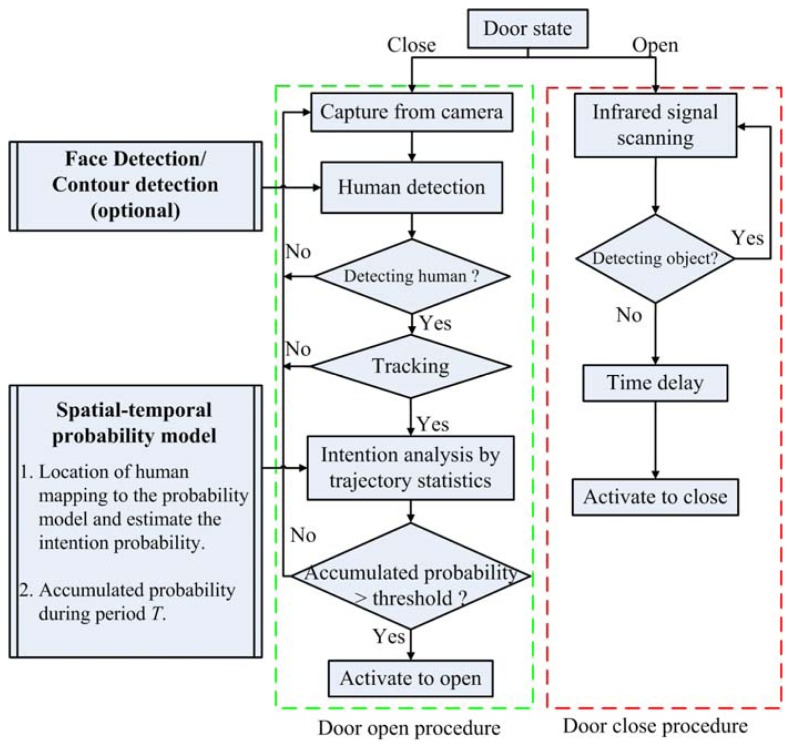
System flowchart.

The proposed innovative door control system is mainly based on human detection and intention analysis. The first part involves face detection or contour detection that identifies whether the detected object is a person, while the latter includes trajectory tracking and statistical analysis for intention estimation. A flowchart of the control procedures is shown in Figure 1.

It is known that face detection with reasonable detection rates had been well developed in the literature and has the closest relevance to human characteristics, thus is good for people identification. In short, once a person is detected in the region of interest (ROI), the trajectory of his face can be tracked and then analyzed by a statistical analyzer to calculate the corresponding cumulative probability, and will be used as the estimation of the intention.

On the other hand, in the door-closing procedure of Figure 1, the door is closed if no object is detected in the passage on both sides of the door. Overall, processes of the state transition, human detection, intention analysis, and the theoretical performance evaluation are described in the following subsections.

### 2.1. State Transition

**Figure 2.**
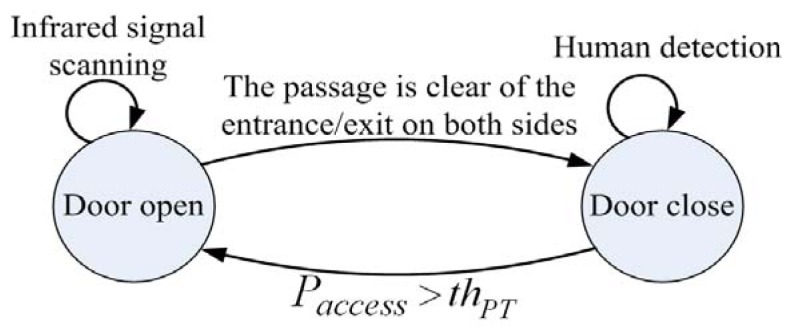
The state transition diagram of the system.

The state diagram of the proposed accessing control system, which includes opening and closing actions, is depicted in Figure 2. The door open process identifies the detect targets as human by face/contour detection, and calculate the door access intention probability, called *P_access_*, by analyzing the corresponding trajectory. Once the *P_access_* is greater than a threshold *th_PT_*, the door is opened accordingly. On the other hand, an infrared sensor is added to make sure that nobody is passing or stayed before door closing, and then activate close action when the entrance/exit is clear for safety.

### 2.2. Human Detection

In recent years, human detection techniques, especially those implemented by face detection strategies, have been successfully applied to many consumer products such as digital cameras, smart phones, or surveillance systems for detecting people [1,2]. In the proposed system, although both face detection and contour detection are adopted for human identification, the face detection is used as the primary solution and the contour detection based on head and shoulder shape, similar to Reference [3], is left as the optional solution for the application which has special privacy considerations. Thus, human detection function is represented by face detection instead of contour detection in the following text. Researches of the face detection can be categorized into four types [4], namely, the knowledge-based, the feature-based, the template matching-based and the appearance-based methods.

In the first method, the face is identified by face features constructed by relative locations and distances of eyes, nose, and mouth [5]. The detection rate is strongly affected by the orientation of the face.

For feature-based approaches, face features are constructed from the statistics of the locations and the distances between the facial organs [6–9]. A face is detected by matching the regions with similar features and skin color information. The detection rate of this method is better than the previous one, but the performance may easily be influenced by the noises, shadows, and lighting conditions [4].

The template matching methods use predetermined facial template for matching. A face is identified once the Euclidean distance between the ROI and the template is less than a certain threshold value [10]. This approach is easy to implement but is not versatile for various types of face geometry.

In the appearance-based methods, statistical analysis and machine learning techniques are used to find the best features of faces [11–13]. Although longer learning time and more samples are needed in the training process, these methods have high detection rate and low processing time and are more practical in real time applications [14] hence will be adopted in our study.

The appearance-based methods can be divided into three types: linear/nonlinear projection, neural network, and Gabor filter/Haar-like feature selection. For example, the principal component analysis (PCA) [15], linear discriminate analysis (LDA) [16], and independent component analysis (ICA) [17] are popular projection based methods in which features extracted from face images are projected into a lower dimensional feature space where the most significant feature vectors are selected. Thereafter, an input sample vector with distance (e.g., Euclidean distance) shorter than a given threshold will be determined as a face image. Basically, performance of this type depends mainly on appropriate distance measurement. In contrast to the projection scheme, the neural network scheme uses huge face image samples for training (such as back propagation network), and then uses the trained network as classifier to detect human faces in the input image [11].

The Gabor filter method constructs a Gabor feature mask from input face sample images and uses it as face discrimination criterion. Although it has high detection rate and little influence by background illumination, the high computation complexity makes it be less practical [18]. Viola *et al.* proposed an Adaboost structure to overcome this drawback [12]. They use a Haar-like mask to train the face database, and determine the spatial feature masks of the face as weak classifiers. These weak classifiers are further cascaded (boosting) as a strong classifier that becomes a good face detector. Although the training process is time consuming, the Adaboost scheme is high in both detection rate and detection speed than neural network approach [12–14] thus is much practical for implementation in embedded system hence is used in the proposed system.

### 2.3. Intention Analysis

The intention analysis also includes two parts: trajectory tracking and estimation of the probability to access the door. The trajectory tracking is realized by comparing the overlapping area of the face images at two consecutive time instants against a threshold value. After that, people's intention of accessing the door is estimated by analyzing the probability distribution of movement in both vertical and horizontal directions. The trajectories of movement in different directions from different locations are recorded first to establish the distribution of ROI. This probability value is then used to calculate the corresponding cumulative density function corresponding to the trajectory to determine whether or not the person has intention to access the door.

#### 2.3.1. Face Tracking via Spatial-Temporal Property

For the commonly used frame rate greater than 15 frames/s, the overlapped part of the two face blocks at two consecutive time instants along the trajectories to the door would be large and the displacement between the block centers $\left(x_{c}^{t-1},\;y_{c}^{t-1}\right)$ and $\left(x_{c}^{t},\;y_{c}^{t}\right)$ is assumed to be small. Thus, algorithm 1 is used to filter out the persons who seems having no intention to access the door.


**Figure 3.**
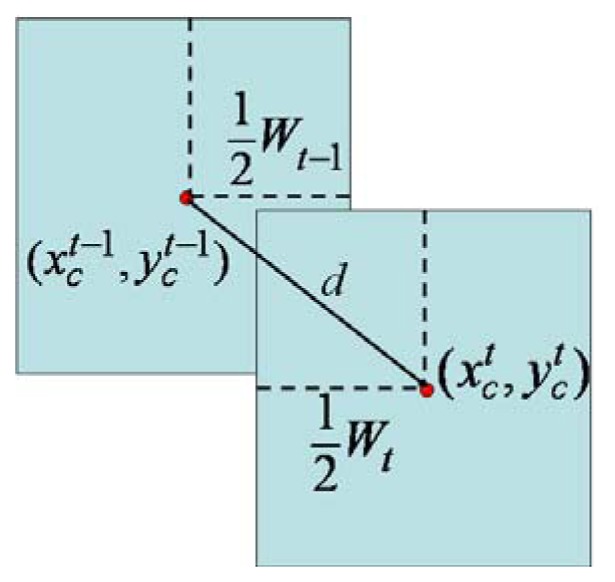
The spatial-temporal property of a moving face.

***Algorithm 1***
If $(d=d\sqrt{d_{\text{yx}}^{2}+d_{y}^{2}}<{\text{th}}_{d}),d_{y}=\left|y_{c}^{t}-y_{c}^{t-1}\right|$then, the two face blocks match.Else, remove the block at time t−1.

where $d_{x}=\left|x_{c}^{t}\;-\;x_{c}^{t-1}\right|,\;d_{y}=\left|y_{c}^{t}\;-\;y_{c}^{t-1}\right|$ and *th_d_* is a given threshold value which is assigned to be the width of a nominal human face *W_t-1_* in our research as shown in Figure 3.

#### 2.3.2. Intention Analysis by Trajectory Statistics

If a person within the ROI has intention to access the door, either the trajectory is characterized by continuous movement toward it or the person's face persists in the ROI for a certain period of time. Thus, the intention can be modeled by first recording the 2D trajectories in front of the door then taking projections along the directions vertical and parallel (or horizontal) to the door and finally conducting curve fitting by appropriate probability density functions (pdf) for the projection curves.

**Figure 4.**
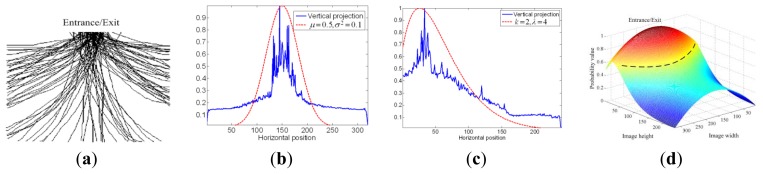
The trajectories and the pdfs (**a**) Trajectories approaching the door (**b**) Vertical projection of the trajectories (blue) and the corresponding pdf (red) (**c**) Horizontal projection of the trajectories (blue) and the corresponding pdf (red) (**d**) Probability table for the joint pdf.

A set of field recorded trajectories approaching the door is shown in Figure 4(a). Note that a person with the intention to access the door will move toward the door, the closer to the door the higher probability that the target has intention to access the door.

Vertical and horizontal projections are conducted to obtain the corresponding 1D histograms *s_v_* and *s_h_* for *x_c_* and y*_c_* respectively, and is indicated by the blue lines in Figure 4(b,c).

Note that the projection *s_v_* looks like a normal distribution while *s_h_* is similar to a non-central Chi-square-distribution as indicated by the red dotted lines in Figure 4(b,c). Thus, we have:

(1a) $$s_{v}\left(x_{c}^{\prime }\right)=\frac{1}{\sqrt{2\pi \sigma^{2}}}e^{\left(-(x_{c}^{\prime }-\mu )/2\sigma^{2}\right)}$$

(1b) $$s_{h}(y_{c}^{\prime };k,\lambda )=\frac{1}{2}e^{-(y_{c}^{\prime }+\lambda )/2}{\left(\frac{y_{c}^{\prime }}{\lambda }\right)}^{k/4-1/2}I_{k/2-1}(\sqrt{\lambda y_{c}^{\prime }})$$

where *x_c_* = *x_c_*/*I_W_* and *y_c_* = *y_c_* × *24*/*I_L_* represents the normalized coordinates, which *I_L_* and *I_W_* is the length and width of the image respectively. To enhance the difference of the probability value at different locations, *s_v_* and *s_h_* is normalized to range from zero to one. Nonlinear least squares curve fitting [19] is then used to estimate the parameters of the pdf for *s_v_* and *s_h_*.

It is assumed, in this paper, that we have *μ* = 0.5 at the center point of the entrance image. It seems once a person has intention to go through the entrance, the moving trajectory is getting together toward the entrance. Thus, the width of *s_v_* increases (it means the variance *σ^2^* of *s_v_*, increase also) in proportion to the size of door (*DS*). On the other hand, parameter *λ* of *s_h_* also increases when *D*, the distance between a pedestrian to the entrance, get increase. That is, *σ^2^* and *λ* can be obtained by:

(2a) $$\sigma^{2}={\left(DS/M\right)}^{2}$$

(2b) λ=D

where *M* denotes the width of ROI. To collect more trajectories for further statistical analysis, *σ^2^* is multiplied by a factor *k* in this paper.

The associated joint probability *p*(*x_c, _y_c_*) is evaluated by means of the inclusion-exclusion principle [19] given in Equation (3), based on which a 3D probability table (PT) is constructed to simplify the computation as shown in Figure 4(d) for a nominal image of size 320 × 240:

(3) $$P\left(x_{c}\;,\;y_{c}\right)=s_{v}\left(x_{c}\right)+s_{h}\left(y_{c}\right)-s_{v}\left(x_{c}\right)\times s_{h}\left(y_{c}\right)$$

To determine whether or not a person has intention to go through the specific entrance/exit we use a sequence of *T* images after a human face is detected in the ROI and calculate the probability of the person's intention to access the door *P_access_* using Equation (4):

(4) $$P_{\text{access}}=\frac{1}{T}\sum_{i=1}^{T}P\left(x_{c}^{i}\;,\;y_{c}^{i}\right)$$

If the face stays long enough during the time interval *T*, the value of *P_access_* will be high. An average value *μ_PT_* of the joint pdf is adopted as the threshold *μ_PT_*, and is defined as:

(5) $$\mu_{\text{PT}}=\frac{1}{PT_{L}\times PT_{W}}\sum_{i=0}^{PT_{L}-1}\sum_{j=0}^{PT_{w}-1}\text{PT}\left(i,j\right)$$

where *PT_L_ and PT_W_* represents the length and width of *PT* respectively, while *PT*(*i,j*) denotes the value of *PT* at (*i,j*) coordinate.

It is observed that if one go through the entrance, the accumulated probability of his trajectory in PT region (the black dotted line in Figure 4(d)) will be greater than *μ_PT_* within *T* interval. Therefore, *μ_PT_* is used, in this paper, as the threshold *th_PT_* to determine whether one has intention to go through the entrance or not. That is, if *P_access_* is greater than *μ_PT_*, the “opening” command is activated.

### 2.4. System Performance Evaluation

The performance of the proposed system is evaluated by both detection rate and false rate *FR_tot_*. In this paper, the face detection is implemented, based on the Viola method [12], by a 10-layers cascade boosting structure. In each layer, the face detection rate is set to be greater than 0.99 at training stage. Thus, the theoretical overall face detection rate is about 0.99^10^ ≈ 0.9. On the other hand, the false rate *FR_tot_* can be defined by *FR_tot_* = *FAR* + *FRR*, where *FAR* and *FRR* are the false acceptance rate and the false rejection rate, respectively. In general, since a non-human face being erroneously identified as a human face will be filtered in the subsequent tracking procedure hence will not cause any false activation of the door, the *FAR* can be ignored and the only false rate that needs attention is *FRR*, or *FR_tot_* = *FRR*. From the experiments, it is observed that the rate of falsely activating the door is below 2 in one million times, compared with the traditional systems that assume nearly 1 out of 5 or 20% of false activation.

#### 2.4.1. False Rejection Rate of the System

The proposed method accumulates the number of the same face images detected during time interval *T* to determine the intention of the target and decides positively if it is greater than *th_PT_*. Thus, the more the face images detected inside the dotted region (Figure 4(d)), the higher the probability that the target has intention to access the door and likewise for the miss. In other words, the more the missed face images, the higher the *FRR*. Thus, *FRR* can be formulated by Equation (6):

(6) $$\text{FRR}=\sum_{i=\text{NF}}^{\text{NT}}C_{i}^{\text{NT}}P{\left(M_{F}\right)}^{i}\times {\left[1-P\left(M_{F}\right)\right]}^{\text{NT}-i}$$

where *NT* = *R_D_* × *T* and *NT* = *RF* × *th_PT_* are the total number of images and the number of face images corresponding to the threshold *th_PT_* to activate the door during time interval *T*, respectively, *R_D_* is the rate of processing a face image, which is five frames/second in our system, and *P*(*M_F_*) is the probability of miss, as shown in Equation (7):

(7) $$P(M_{F})=P(\text{MR})\times P({\text{th}}_{\text{PT}})$$

where *P*(*th_PT_*) denotes the ratio of area whose value is greater than *th_PT_* over the total area of *PT*, which is 0.54 in our experiments, and *P(MR)* is the miss rate of face detection defined by *P(MR)* = *1*−*P(DR)*, with *P(DR)* being the detection rate, which is 0.9 in our system, as described previously. The theoretical estimation show that if *T* = 2 s, *FRR* will be close to 2.4 × 10^−5^, but if *T* = 3 s, *FRR* will be 3.9 × 10^−8^ and lower.

## 3. Experiment Results and Discussion

**Figure 5.**
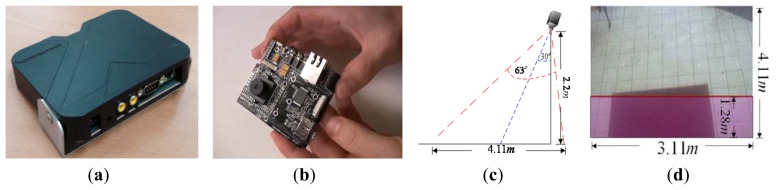
Prototype of the proposed system and the installation parameters illustration. (**a**) and (**b**) are the system prototype appearance and designed hardware platform, respectively. (**c**) and (**d**) illustrate the system installation example and the area for infrared signal scanning.

The proposed system can determine the user's intention and provide access to a door by detecting his position and tracking his trajectories of movement in the image sequence. To implement the system, a DSP based multimedia module, TI DM368, is selected as the hardware platform. The algorithms developed were programmed in C and executed under the Linux platform. The specifications of hardware and software are as follows: an ARM9 CPU with a speed of 900 MHz with embedded processing algorithm is used. The system prototype and the DSP platform board are shown in Figure 5(a,b). The image sensor resolution is 500 M and the test image size is 320 × 240. The other installation parameters are described as follows: the camera is installed on a wall at 2.2 m high and 30° tilt. The size of ROI is 3.11 m (width) and 4.11 m (distance to door) respectively, as shown in Figure 5(c). The width of door is 1.6 m. Thus, according to Equation 2(a,b), *σ^2^* of *s_v_* and *λ* of *s_h_* can be calculated as *σ^2^* = 0.26 and *λ* = 4.11, respectively. The infrared signal scanning in ROI is indicated by the red area, as shown in Figure 5(d). The scanning width and distance are 3.11 m and 1.28 m (red line to door), respectively.

**Figure 6.**
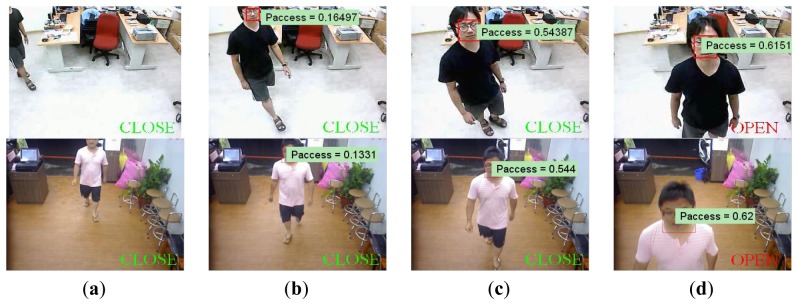
Opening activated by single person in lab (**first row**) and restaurant (**second row**) scenes: The cumulative probability increases if a person is continued walking toward the entrance thus the face is detected. Once the cumulative probability is greater than the threshold value, the opening action is triggered as shown in (**d**).

**Figure 7.**
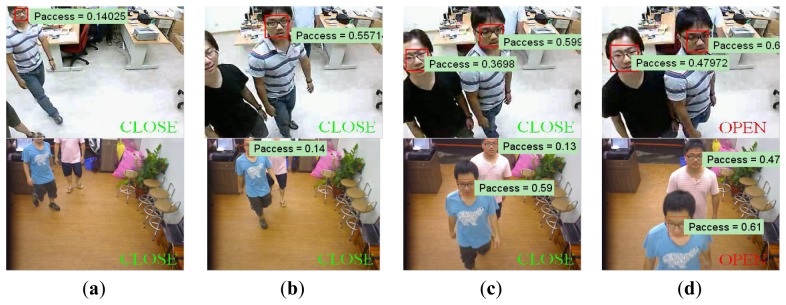
Opening activated by multiple persons in lab and restaurant: The cumulative probability values for all detected faces are calculated independently. Thus, as long as one of the cumulative probability values exceeds the threshold, the door will be opened as in (**d**).

**Figure 8.**
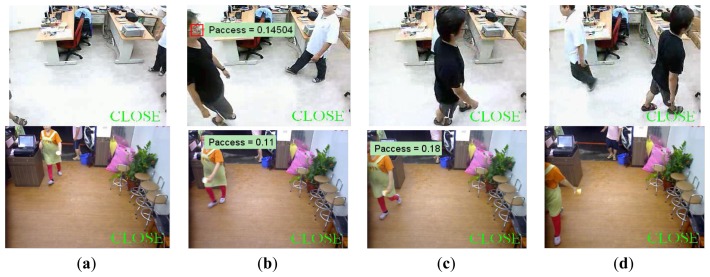
Illustration of “just passing by” case in lab and restaurant: It is observed that although a person is detected in ROI, the door is not opened since the person is just passing by the entrance, thus the cumulative probability does not exceed the threshold value.

Demonstrations of processing results under different cases, including single entering, multiple entering, and just passing through, are shown in Figures 6, 7 and 8.

**Figure 9.**
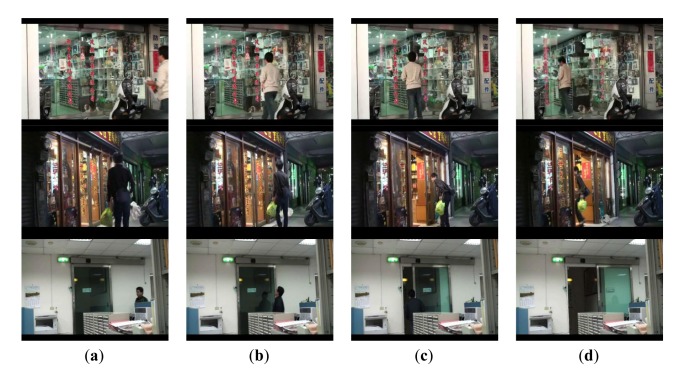
Illustrations of success door activation by person with accessing intention at 3 business places. The first row: electronic materials store (day). The second row: wine store (night). The third row: business office (indoors).

**Figure 10.**
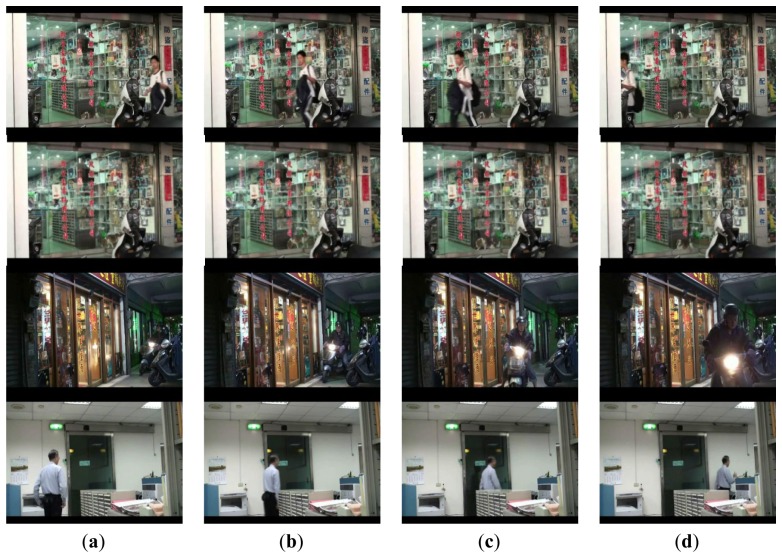
Illustrations of correct activation rejection at the same places. The first and second rows show the cases of a human passing by and stay of a lying dog, respectively. The third and fourth rows show the passing by conditions of a motorcycle and a human respectively.

In the case of single person, it is observed in Figure 6(b–d), that the cumulative probability increases if a person continues walking toward the entrance so the face is detected. Once the cumulative probability is greater than the threshold value, the opening action is triggered as shown in Figure 6(d). In the multi-persons case of Figure 7, cumulative probability values for all detected faces are calculated independently. Thus, as long as one of the cumulative probability values exceeds the threshold, the door will be opened as shown in Figure 7(d). Finally, in Figure 8, it is observed that although a person is detected in ROI, the door is not opened since the person is just passing by the entrance, thus the cumulative probability does not exceed the threshold value. To evaluate the performance of the proposed system, five different scenes including one lab and four business places are used, while the test condition includes day, night, indoor, and outdoor conditions. The four business places include two shops, one restaurant, and an office. Moreover, to illustrate the effect of the system, three of these places are recorded by a side view camera set and are shown in Figures 9 and 10.

By observing the process from the embedded camera at the lab and restaurant places which are shown in Figures 6, 7 and 8, it is also observed from Figures 9 and 10 that the door is correctly activated by the pedestrian who has intention to access, it but kept closed while he is just passing by or the image object is not a real person. Interested readers can access the recorded videos [20] to see the experimental process. The false rate result collected from the above places is presented in Table 1.

It is observed that the face detection function is hardly affected by the following cases such as wearing glasses, mask, hat, *etc.* The collected data, in 253 trials at five different locations, shows that the correct opening rate within the default 2 s is 0.996 (252/253) while the incorrect action number is only 1. It is noted that although the *FAR* (0.004) is slightly higher than the theoretically predicted number, for the only one failure case, however, the door is still opened but just delayed for a short time (the response time is 4 s). Moreover, an interesting phenomenon is observed that although some people with access intention are walking dejected/passing with heads down thus no face is detected, finally they stop behind the close-door and look up for something to open the door. These actions, however, reveal their faces which are detected by the camera and finally the opening action is activated after a few seconds delay. Therefore, there exists zero case when a person wanted to enter was rejected and it can reject the false opening actions for people passing, staying, and non-human cases, no matter in the day, night, indoor, or outdoor conditions.

## 4. Conclusions and Future Works

In this paper, an automatic door control system is implemented on a DSP platform. The system can first identify a target as person by face detection, and then analyze the path trajectory to determine whether the person has intention to access the door or not, thus to control the door accordingly. It is noted that the system has advantages of low false rate (near 0%), high correct activating rate (99.6%), and short response time (within 2 s) from detecting the target, confirming his intention, to activate the door opening. Moreover, via statistical analysis on detected face in consecutive time sequence, case of passing persons with missing face can still be confirmed within 4 s thus to activate the door correctly, as shown in Table 1.

To sum up, the proposed method builds up the statistic model of moving trajectories in ROI first, and the corresponding probability of a face at certain location can be obtained by lookup table. If the average probability of face trajectory is greater than *th_PT_* within *T* period, the person is said to have intention to go through the entrance. That is, region within dotted line as shown in Figure 4(d), whose *pdf* is greater than *th_PT_*, is viewed as key region while determining one's intention. If the face trajectory locates mostly within the key region, the person is said to have intention to go through the entrance. According to Equation (6), if *th_PT_* is set to be 0.8 (or 0.2) and *T* is set by 2 s, the *FR_tot_* will become 0.18 (or 6.5 × 10^−8^). The response time to confirm one's intention will increase when *th_PT_* increase, while lower *th_PT_* value will result more false opening actions. It is observed, by our experiments, that if *th_PT_* is set to be the average of ROI, said *μ_PT_*, the *FR_tot_* is about 6.3 × 10^−5^ and is suitable for most application cases.

The proposed intelligent control system, compared with traditional ones, not only reduces the false action rate, but offers extra power saving benefits. For example, less false door activation reduces the energy exhaust of air conditioners as well as the door driver. Moreover, combining face recognition as well as behavior analysis algorithm into the built-in camera and DSPs module, it is possible to add disaster or crime prevention functions that thus can be applied to surveillance applications. To sum up, although the cost is somewhat higher than that of traditional systems, the proposed system should be viewed as, instead of an accessing control system only, a general purpose intelligence video surveillance (IVS) system as long as the detection function is replaced according to the related applications, for example, to detect the car, motorcycle, or detect the static object application in home healthcare [21] or pedestrians in a forbidden region by adopting the corresponding detection function and 3D probability model. Some of the extended functions such as plate detection, pedestrian detection, and flood detection, had been successfully examined in the proposed platform [22,23], while other functions with huge complexity, e.g., fire detection, can also be fulfilled using a similar hardware structure but replacing a faster DSP module. That is, the proposed system is designed not only as an access control system but also a watch dog in front of the entrance, thus, the CP value of the proposed system is much greater than that of the existing systems.

## Figures and Tables

**Table 1.** The Experimental Results of False Rate.

**Results/Location**		**No. of Persons**	**Failure Count**	**Detection Rate**
Real Test	Electronic materials store (Banqiao city)	52	1	99.5%
	Wine store (Yingge city)	28	0	
	Office (Yangmei city)	45	0	
	Restaurant (Xindian city)	72	0	

Lab. Test	Lab. (Banqiao city)	56	0	100%

Total		253	1	99.6%
